# Contribution of groundwater iron to cooked rice and the implication on the recommended iron intakes: a cross-sectional study in Bangladesh

**DOI:** 10.1017/jns.2025.10063

**Published:** 2025-12-22

**Authors:** Nobonita Saha, Sabuktagin Rahman, Towhid Hasan, Sneha Sarwar, Marjia Sultana, Abu Ahmed Shamim, Nazma Shaheen

**Affiliations:** 1 Institute of Nutrition and Food Science, University of Dhaka, Dhaka, Bangladesh; 2 Department of Public Health, American International University-Bangladesh, Khilkhet, Dhaka, Bangladesh; 3 Department of Food Technology and Nutrition Science, Noakhali Science and Technology University, Noakhali, Bangladesh; 4 BRAC James P. Grant School of Public Health, BRAC University, Dhaka, Bangladesh

**Keywords:** Groundwater iron, Cooked rice, Iron retention, Bangladesh, AAS, Atomic Absorption Spectrophotometry, BRRI, Bangladesh Rice Research Institute, DO, Dissolved Oxygen, DRI, Dietary Reference Intakes, DOM, Dissolved Organic Mass, EAR, Estimated Average Requirement, Hb, Hemoglobin, ID, Iron Deficiency, IDA, Iron Deficiency Anemia, INFS, Institute of Nutrition and Food Science, NPNL, Non-pregnant and non-lactating women, RDA, Recommended Dietary Allowance, SE, Standard error, WDR, Water draining rice, WSR, Water sitting rice, WHO, World Health Organization

## Abstract

Groundwater iron varies geographically and iron intake through drinking water can minimise iron deficiency (ID). Rice, a major share of daily meals (∼70% of total energy) in Bangladesh, absorbs a substantial amount of water. This study aimed to estimate the contribution of groundwater iron entrapped in cooked rice and its implications on the recommended iron intake. A cross-sectional study was conducted among 25 households, selected by the iron content of their drinking groundwater source in Sirajganj district, Bangladesh. Each household pre-supplied with 600 g of raw rice (300 g for each cooking), was instructed to cook ‘water-draining rice’ (WDR) and ‘water-sitting rice’ (WSR). Using atomic absorption spectrophotometry, iron content in filtered and non-filtered water was measured as 0.4 ± 0.2 mg/L and 6.1 ± 2.0 mg/L, respectively. After adjusting for water filtration, the weighted mean of total iron content in WDR and WSR was 6.18 mg and 5.70 mg, respectively. Assuming the average rice intake, iron content in WDR and WSR fulfilled approximately 98.15% and 90.62% of the average requirement for non-pregnant and non-lactating women (NPNL). The water-entrapped iron in cooked WDR and WSR fulfilled about 23.77% and 20.4% of Recommended Dietary Allowances, and 52.83% and 45.30% of Estimated Average Requirements, respectively in NPNL women, suggesting that groundwater entrapped in cooked rice is an influential dietary iron source. The substantial amount of iron from cooked rice can make an additional layer to the environmental contribution of iron in this setting with the potential to contribute ID prevention.

## Graphical abstract

**Figure:**
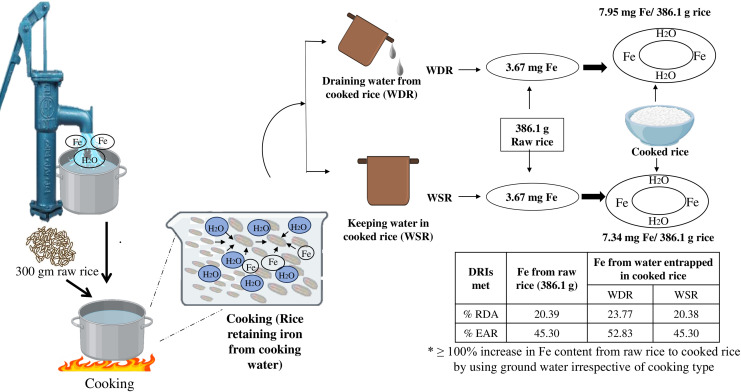

## Introduction

Although iron is the second most abundant metal and is highly bioavailable in nature, iron deficiency (ID) is the world’s most common micronutrient deficiency.^(1)^ ID develops from insufficient iron intake or disrupted utilisation of available iron, culminating in iron deficiency anaemia (IDA).^(2,3)^ IDA is a global public health problem predominantly in developing countries.^(4)^ Globally, in 2011, the prevalence of IDA among preschool children, pregnant women, and non-pregnant and non-lactating women (NPNL) was about 42.6%, 38.2%, and 29%, respectively.^(4)^ According to the National Micronutrient Survey 2011–12, the national prevalence of ID in Bangladesh in preschool children, school-aged children and NPNL women is 10.7%, 3.9–9.5% and 7.1% respectively which is surprisingly low.^(5)^

The attribution for such a low prevalence of ID is the bioavailable iron from groundwater in Bangladesh. About 76.8% of people depend on tube wells for drinking water^(6)^ and approximately 6.71 L/person/day groundwater is utilised for cooking.^(7)^ The majority of areas of Bangladesh contain high iron levels (median 1.1 mg/L, maximum 61mg/L) in groundwater.^(8)^ Several studies have provided evidence of a clear positive association between drinking groundwater and inflammation-adjusted ferritin in populations.^(9–12)^ While iron is essential for vital functions of the body,^(2)^ excess iron is linked with unwanted health effects, such as nausea, vomiting, loose stools and abdominal discomfort.^(13,14)^ Bangladeshi traditional diet is predominantly cereal-based and rich in phytate, which causes poor absorption of bi-valent minerals, such as iron. At another level, a recent study has shown a weak non-significant effect of dietary iron on the total serum ferritin in the backdrop of high serum ferritin status, attributed to the reserve developed by groundwater iron.^(15)^

The groundwater iron varies nationwide and a recent study has shown that even at a low level of groundwater iron (<0.8 mg/L) helps to maintain higher Hb concentration in young children at the non-anaemic level,^(16)^ suggesting a protective role of groundwater iron. This perspective establishes the powerful role of drinking water iron in maintaining the iron status in the Bangladeshi population. Supporting this context, this study explores an extension of environmental iron sources in Bangladeshi population. Rice grains absorb water during cooking and increase the volume by 2.5 to 3 times depending on rice varieties and cooking procedure. The yield factor of WSR (BR-28) is 3.04, representing high water absorption during cooking.^(17)^ It suggests that 100 g of rice grains absorb ∼ 200 ml of water. In Bangladesh the cooked rice is consumed approximately 328.9 g per day by an adult.^(6)^ The intent of the study is to quantify the contribution of the iron entrapped in the water of the cooked rice to the dietary recommended intakes. This analysis is essential on the backdrop of sufficient iron status in Bangladeshi population, largely attributed to the iron from the drinking groundwater. There are several supplementation programmes for iron, such as iron-folic acid/multiple micronutrient supplements for women of reproductive age, micronutrient powder for children, and iron-fortified rice. Multiple channels of iron intake may lead to potential iron excess and iron-induced side effects.^(13,14)^ Hence, the present study explored the amount of iron consumed from rice-entrapped groundwater and assessed its potential contribution to dietary reference intake. The results of the present study may add to the knowledgebase, of a novel, habitual and culturally integral iron source thus may inform the need for adjustment of the multiple supplemental/fortified sources for optimum iron intake; and therefore, possibly can avoid the excess.

## Methodology

### Study site

**Figure 1.**
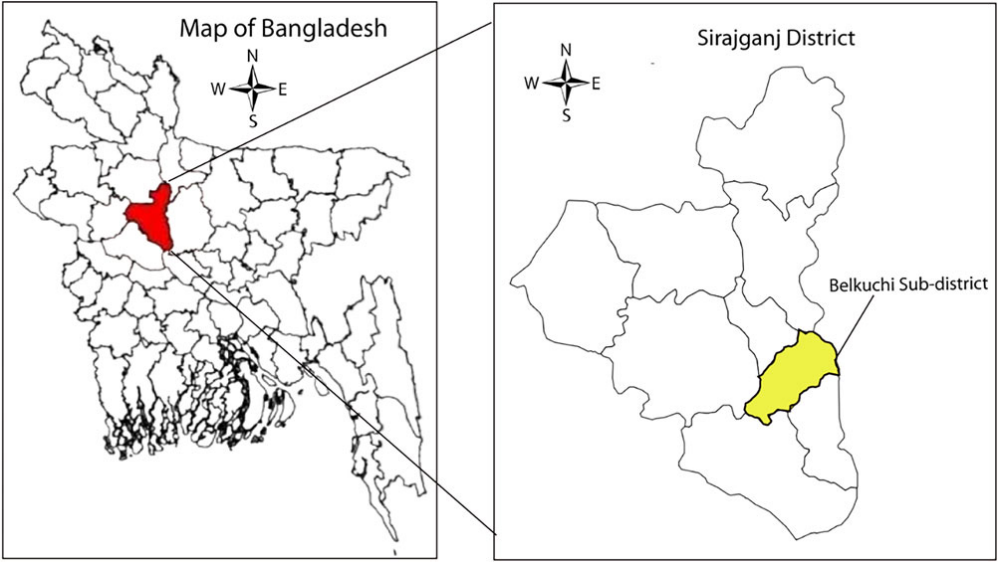
Study site — Belkuchi, a north-western sub-district of Bangladesh (24.2917°N, 89.7000°E).

A cross-sectional study was conducted at Belkuchi Upazila (24.2917°N, 89.7000°E) of Sirajganj district of Rajshahi division, in the northwestern part of Bangladesh (Figure 1) from May to June 2022. The study site was selected because as per the British Geological Survey 2001^(8)^ the area belonged to high groundwater zone (≥ 2 mg/L) using the cut-off the water iron concentration suggested by the World Health Organization, 2008.^(18)^

### Sample size and sampling strategy

The purposive sampling was employed to select 40 households with tube wells. The inclusion criteria of the households were as follows (a) having a source of drinking and cooking water from a tube well and (b) belonging into a group of pre-determined classifications of groundwater iron concentration (Table 1).

**Table 1.** The selection of the tube wells

Group depending on groundwater iron concentration	Number of tube wells identified by spot kit^a^	Number of tube wells selected for data collection^b^
Group 1: low iron concentration (0–<1 mg/L)	8	5
Group 2: medium iron concentration (1–2 mg/L)	8	5
Group 3: high iron concentration (3–10 mg/L)	8	5
Group 4: very high iron concentration (>10 mg/L)	8	5
Group 5: using filtered water	8	5
Total	**40**	**25**

a Eight tube wells are selected purposively to include in each of the 5 groups.

b Five tube wells are randomly chosen from the 8 tube wells that belong to each of the groups.

Groundwater iron content of their tube wells was measured by a portable ‘spot kit’ device (Handheld Colorimeter, HI721 Checker®) HC, Hanna Inc. Woonsocket, RI, USA). The device has been validated for the measurement of groundwater iron concentration against a gold standard measure, the atomic absorption spectrophotometry (AAS).^(19)^ The tube wells were hand-pumped for 3–5 minutes to collect water from deeper aquifers to avoid the formation of ferric precipitates which may build up in the inner walls of the wells. Thereafter, the device was ‘zeroed’ with the vial inside. Next, in the manufacturer-provided test beaker, a 10 mL water sample was drawn and combined with the supplied phenanthroline reagent; and a thorough mixing was done. The test beaker with the reagent mixed sample water was placed in the device. After pressing the button, the result was displayed in numerical digits. This test kit estimated an iron concentration up to 5 mg/L. The test was repeated with two-, five- or ten-fold dilutions with distilled water if the concentration of iron exceeded the reference. The final reading was adjusted by multiplying the corresponding units of dilution. Depending on the iron content (low to very high), five groups were considered and eight households were purposively considered in each group. Subsequently, five households were randomly selected from each group to include in the final study (samples *n* = 25) (Table 1).

### Data collection procedure

A structured questionnaire was developed to collect information on socio-demographics, practice of water filtration and the rice-cooking practices. The questionnaire was pretested and standardised according to the study objective. The participants were the primary caregivers in the households. The participants were required to cook rice by two different methods: WDR and WSR — two common methods of rice cooking in Bangladesh. They were not given recipes for these methods but allowed their usual practice of cooking. For both methods of cooking, they were provided with 600 g of raw rice (300 g for each method). Earlier studies have suggested that the mineral and nutrient properties in rice are influenced by different cooking methods depending on the rice-to-water ratio during cooking.^(20)^ Hence, the participants were asked to cook rice following both cooking methods using the provided raw rice. Among the rice varieties in Bangladesh, BRRI dhan 28 (BR-28) is one of the major varieties which has a high yield potential.^(21)^ It is the dominant variety and commonly consumed in the study area. The BR-28 (10% milled) was procured from the Bangladesh Rice Research Institute (BRRI).

During cooking, the participants were requested to use aluminium pots to minimise external iron contamination. About 50 mL water sample (non-filtered) from each tube well was collected by the data collectors in polyethylene falcon tubes after hand-pumping the tube wells for 3–5 minutes. Similarly, about 50 mL of filtered water sample was also collected from the households that used filtered groundwater for cooking. The falcon tubes were previously rinsed with deionised water. The collected water samples were immediately mixed with 2 mL nitric acid to avoid any precipitation of iron. The data collectors observed the cooking process. The cooking time, frequency of washing rice before cooking, amount of groundwater used for cooking and amount of discarded gruel for each cooking method were recorded by the data collectors. After the end of cooking process, approximately 90–100 g of cooked rice was collected separately for each cooking method using a wooden spatula in a polyethylene plastic jar pre-washed with deionised water and air-dried. Cooked rice samples were set to cool before closing the lid of the jar and immediately stored in a refrigerator (4 °C) to avoid any food spoilage. A total of 50 cooked rice samples (25 WDR and 25 WSR) were collected. The cooked rice samples were transferred to a refrigerator (4 °C), and the water samples were stored at room temperature at the Institute of Nutrition and Food Science (INFS), University of Dhaka, for further analysis. Finally, the raw and cooked rice samples were sent to the Environmental Science Laboratory at the International Centre for Diarrheal Disease Research, Bangladesh (icddr,b), Dhaka, to measure the iron concentration by atomic absorption spectrometry (AAS) and the water samples (filtered and non-filtered) were sent to the laboratory of the Department of Geology, University of Dhaka to estimate the iron concentration by AAS.

### Calculation

There are a number of key variables; for example, volume of water absorbed in cooked rice [A], weight of the cooked rice [B], weight of the raw rice grains [C], volume of water used to cook rice [D], volume of water vaporised during the cooking [E], volume of gruel discarded during the cooking [F], the concentration of iron in filtered or non-filtered water [G], Iron content in the entrapped water of the cooked rice [H], iron content of the raw rice grains [I] and the total iron content in the cooked rice [J].

- The volume of water absorbed in cooked rice was estimated by subtracting the weight of the raw rice grains from the weight of cooked rice [A = B - C].
- The volume of water vaporised during cooking was estimated — at first, by summing the volume of the discarded gruel and the volume of water absorbed in cooked rice; followed by deducting this combined volume from the volume of water used to cook rice [E = D - (F + A)].
- Iron content in the entrapped water of the cooked rice was estimated by multiplying the concentration of iron in filtered or non-filtered water with the volume of water absorbed in the cooked rice [H = G × A].
- The total iron content in cooked rice was estimated by summing the iron content in the raw rice grains and the content of iron in the entrapped water of the cooked rice [J = I + H].
- The ratio of the weight of the cooked rice [B] to the weight of the rice grains [C] estimated the yield factor for the rice.

Socio-demographic characteristics of the participants, such as age, occupation, education of the respondents, and household heads were obtained. The contribution of the iron from the groundwater entrapped in cooked rice was assessed as percentages relative to the Dietary Reference Intakes (DRI). The DRIs include Estimated Average Requirement (EAR) and the Recommended Dietary Allowance (RDA) for micronutrients. EAR and RDA for non-pregnant non-lactating women aged 19–50 years were considered for comparisons as 8.1 mg/day and 18 mg/day respectively.^(22)^ We used 300 g of rice to estimate the iron content in the rice-entrapped water. However, a recent study reported the mean consumption of raw rice in Bangladesh is 386.1 g.^(23)^ Hence, the iron content in the rice entrapped water was estimated considering both the amounts.

### Statistical analysis

Statistical analysis was performed by Statistical Package for the Social Sciences (SPSS) software (version 26, IBM Company, Chicago, USA). The frequency distribution and percentage were determined for the socio-demographic characteristics of the participants. The normality of the data was checked using histogram and Q-Q plots prior to performing statistical tests. When data were skewed, Mann-Whitney test for two independent groups was used to report the probability of group differences. *p* ≤ 0.05 was considered significant for all tests. Cronbach’s α reliability coefficients for internal consistency of the data were estimated considering the variables — (a) volume of water used to cook WDR, (b) time required to cook WDR, (c) weight of WDR, (d) volume of drained water for WDR, (e) volume of absorbed water for WDR and (f) volume of vaporisation for WDR. Cronbach’s *α* was 0.78, which is indicative of a fair degree of interrelatedness of the variables, thus suggestive of good reliability of the data.

### Ethical approval

An application form filled with information regarding the purpose and rationale of the study, a brief description of the proposed methodology, and procedures for providing feedback to the participants were submitted to the Institutional Review Board of the Faculty of Biological Science, University of Dhaka; and the protocol was approved (reference no: 179/Biol. Sci.). The data collectors described the purpose of the study to the participants and an informed written consent was obtained before data collection.

## Results

A total of 25 households were included in this study. Five tube-wells were from each of 5 groups — 4 groups represented low-to-high concentrations of iron in water and one group represented households using filtered water (Table 1). Respondent was an adult female from each household. The socio-demographic characteristics of the 25 participants are presented in Table 2. The average age of the participants and husbands of the participants were 34.44 ± 13.26 years and 41.92 ± 11.19 years, respectively. Almost all the participants (88%) were housewives. Nearly 40% of the participants’ husbands earned their livelihood through diverse occupations, such as handloom industry, services, garment workers, and driving. In terms of educational status, 68% of the participants and 52% of the husbands of the participants did not complete secondary education.

**Table 2.** Socio-demographic and household characteristics of the participants

Variables with categories	Frequency (*n*)	Percentage (%)
*Age of the participants*		
< 30 years	12	48
30–60 years	10	40
> 60 years	3	12
*Occupation*		
Unemployed/housewife	22	88
Working	3	12
*Education*		
Functionally illiterate^a^	7	28
Incomplete secondary	17	68
Secondary and above	1	4
*Husband’s age*		
< 30 years	0	0
30–60 years	21	84
> 60 years	4	16
*Husband’s occupation*		
Agriculture	6	24
Business	10	40
Others^b^	9	36
*Husband’s education*		
Functionally illiterate^a^	7	28
Incomplete secondary	13	52
Secondary and above	5	20
*Usage of filtered water*		
Yes	5	20
No	20	80

a Functionally illiterate was defined by UNESCO in 1978.^(37)^

b Other occupations of husbands include service, expatriate worker, factory worker, and driver.

Households used the tube well water (a type of piped-well to extract groundwater) for their drinking and cooking either in filtered or non-filtered form. The proportion of the respondents who used filtered water and non-filtered water was 20% and 80% respectively (Table 2).

The mean (± SE) iron concentrations in the filtered and non-filtered water were 0.4 ± 0.2 mg/L and 6.1 ± 2.0 mg/L, respectively. The weighted mean of iron concentration adjusting the filtration status was 4.99 mg/L. The non-filtered water contained significantly higher iron than the filtered water (*p* = 0.021) (Table 3).

**Table 3.** Iron content in the groundwater used for cooking

Variable	Frequency, n	Mean (mg/L)	SE (mg/L)	Weighted mean (mg/L)*	*p*-value**
Non-filtered water	20	6.1	2.0	4.99	0.021
Filtered water	5	0.4	0.2		

SE = Standard error.

* Weighted mean of iron concentration in groundwater accounted for the mean iron concentrations in the filtered and non-filtered water.

** Mann–Whitney test to report the statistical difference of mean water iron concentrations between non-filtered and filtered water.

Table 4 shows the water usage profile for cooking rice and the rice-to-raw rice conversion factors. A significantly higher amount of water was used to cook WDR than WSR (1610 ± 80.4 mL vs. 1008 ± 39.1 mL, *p* < 0.001). The mean weight of cooked WDR was significantly higher compared to WSR (*p* = 0.003). However, no significant differences were found between WDR and WSR regarding the amount of water vaporised (*p* = 0.778). Statistically significant differences were observed between WDR and WSR regarding the amount of water absorbed in raw rice (*p* = 0.003) and rice-to-raw rice conversion factor (*p* = 0.003).

**Table 4.** Water usage profile to cook rice and the rice-to-raw rice conversion factor (*n* = 25)

Variable	WDR	WSR	*p- value*
Volume of water used to cook (ml)	1610 ± 80.4^a^	1008 ± 39.1^b^	< 0.001
Volume of drained water after cooking (ml)	551.5 ± 64.9	–	–
Weight of cooked rice (g)	948.4 ± 32.1^a^	878.6 ± 15.7^b^	0.003
Volume of water absorbed in raw rice (mL)	648.4 ± 32.1^a^	578.6 ± 15.7^b^	0.003
Volume of water vaporised (mL)	411.04 ± 52.4^a^	422.2 ± 34.2^a^	0.778
Rice-to-raw rice conversion factor	3.16 ± 0.1^a^	2.93 ± 0.1^b^	0.003

WDR: Water-draining rice; WSR: Water-sitting rice; Data are mean ± standard error.

ab Different letters in the same row indicate significant statistical differences between WDR and WSR; *p* < 0.001 (volume of water used), *p* < 0.003 (weight of cooked rice), *p* < 0.003 (volume of water absorbed in raw rice), *p* < 0.003 (rice-to-raw rice ratio).

aa Similar letters in the same row indicate non-significant statistical differences between WDR and WSR; *p* > 0.05 (volume of water vaporised).

**Figure 2.**
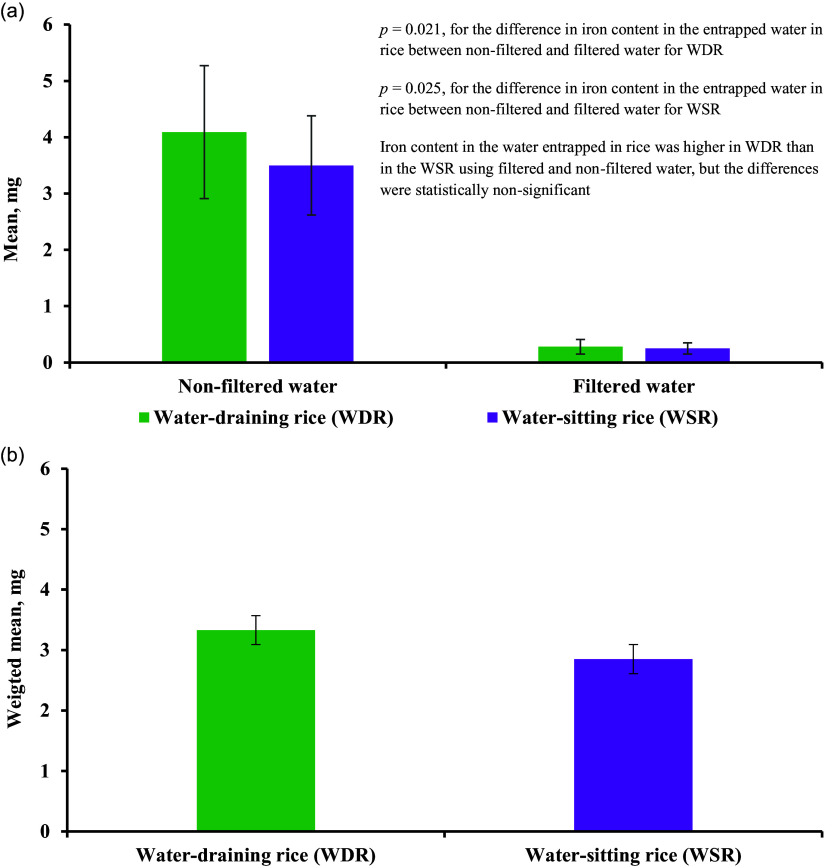
Iron content in the water entrapped in rice during cooking 300 g of raw rice a) mean iron content by cooking type and filtration status b) weighted mean iron content.

**Figure 3.**
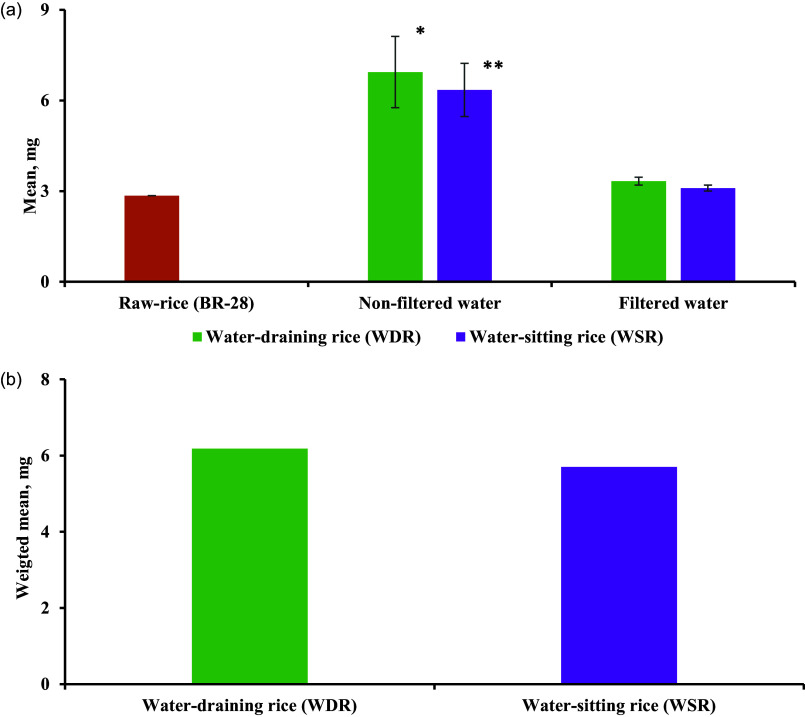
Total iron content in 300 g of water-draining rice (WDR) and water-sitting rice (WSR) — a) mean iron content in raw rice and cooked rice by cooking type and filtration status b) weighted mean iron content adjusted for filtration. *Note*: *Difference in iron content is significantly higher in WDR cooked by non-filtered water than that cooked by filtered water (p = 0.021); **Difference in iron content is significantly higher in WSR cooked by non-filtered water than that cooked by filtered water (p = 0.025).

Figure 2 showed the iron content in the water entrapped in rice during the cooking of 300 g of raw rice. The iron content was significantly higher in the rice-entrapped water using non-filtered water than in filtered water for both WDR (*p* = 0.021) and WSR (*p* = 0.025). The iron content in the rice-entrapped water was higher in the WDR compared to the WSR for both non-filtered and filtered water; however, the difference was statistically non-significant. The total iron content in the raw rice, WDR, and WSR were summarised in Figure 3. The raw rice had significantly lower iron content compared to WDR and WSR, irrespective of filtration (*p* ≤ 0.05). In addition, total iron content was significantly higher while using non-filtered water than using filtered water for both WDR (*p* = 0.021) and WSR (*p* = 0.025) (Figure 3(a)). However, no significant difference was observed between WDR and WSR in the case of iron content in filtered and non-filtered water (Figure 3(a)). After adjusting for the filtration of water, the weighted mean of total iron content in WDR and WSR was 6.18 and 5.70 mg, respectively, with no significant difference (Figure 3(b)**)**.

Table 5 showed the iron content in raw rice, WDR and WSR at two levels of consumption — 300 g and 386.1 g of rice. The table further showed the percent increment of iron content in cooked rice relative to the raw rice at both levels of consumption. Cooking significantly increased iron content. The WDR increased the iron content to 6.18 mg from 2.85 mg in raw rice (116.74% increases) for 300 g rice (*p* < 0.05). Using 386.1 g rice, the WDR increased the iron content to 7.95 mg from 3.67 mg in raw rice (116.63% increases; *p* < 0.05). Similarly, the WSR increased the iron content by 100.1% irrespective of the use of 300 g or 386.1 g rice.

**Table 5.** Iron content in raw and cooked rice and increment of iron content in cooked rice relative to raw rice

Variable	Raw rice	Cooked rice	
		WDR	WSR
Iron content (mg in 300* g)	2.85 ± 0.00^a^	6.18 ± 0.99^a^	5.70 ± 0.75^b^
% increment of iron content in cooked rice relative to the raw rice for 300 g of rice	–	116.74 ± 34.81	100.07 ± 26.39
Iron content (mg in 386.1 g**)	3.67 ± 0.00^a^	7.95 ± 7.08^a^	7.34 ± 5.36^b^
% increment of iron content in cooked rice relative to the raw rice for 386.1 g of rice	–	116.63 ± 35.01	100.00 ± 25.67

%: percentage, WDR: Water-draining Rice; WSR: Water-sitting Rice; Data are mean ± standard error.

*Test amount of rice; **Daily rice consumption per capita per person in rural Bangladesh = 386.1 g.^(23)^

aa Statistically significant differences between WDR and raw rice at *p* ≤ 0.05.

ab Statistically significant differences between WSR and raw rice at *p* ≤ 0.05.

**Figure 4.**
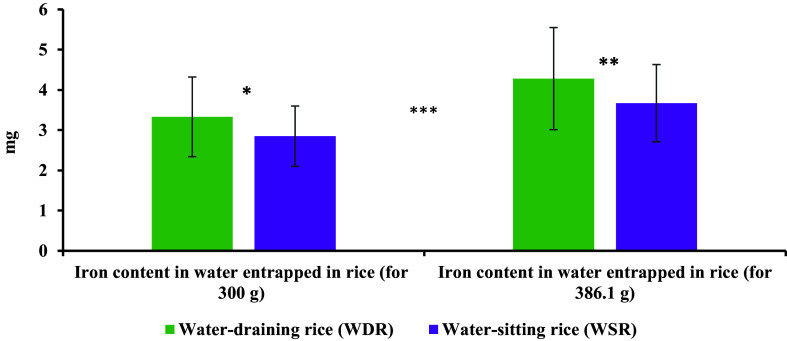
Content of iron in the water entrapped in rice. *Note*: *300 g rice per cooking method was used in the study (test amount); **386.1 g rice is the average intake in non-pregnant and non-lactating women per day;^(23)^ ***Iron content in the water entrapped in rice is slightly higher in WDR than in WSR, but the difference is statistically non-significant.

Figure 4 illustrates the amount of water-entrapped iron in rice. Considering the two different levels of rice consumption (386.1 g and 300 g), Figure 4 highlighted the role of cooking water in enhancing iron content. Iron content in water entrapped in rice was observed to be slightly higher in WDR compared to WSR for both 300 g and 386.1 g amount of rice.

**Figure 5.**
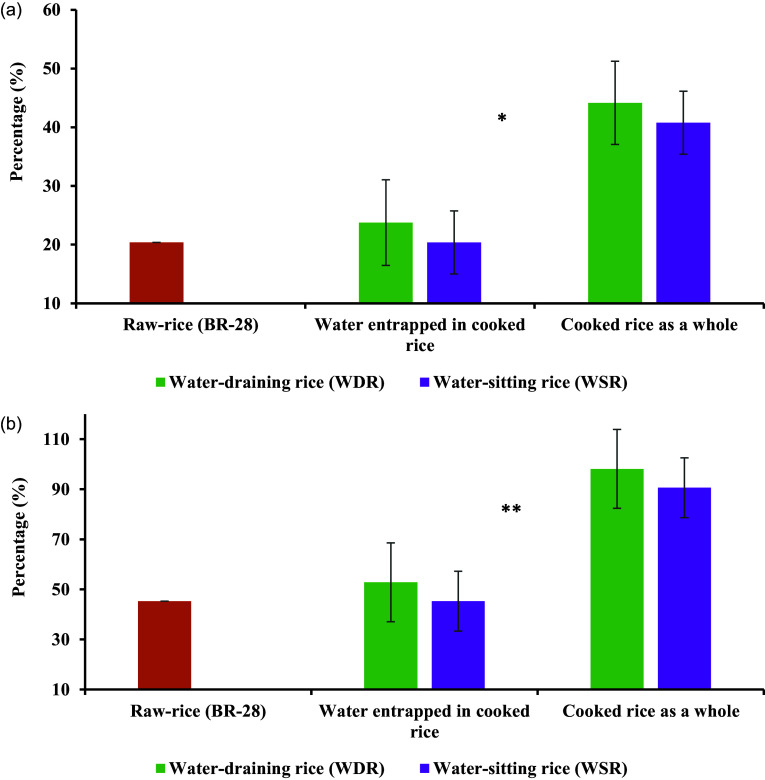
Iron content to potentially contribute to (a) RDA*** and (b) EAR**** by the consumption of 386.1 g rice in non-pregnant and non-lactating (NPNL) women (RDA for NPNL women is 18 mg/day^(22)^ and EAR for NPNL women is 8.1 mg/day.^(22)^ *Note*: *No statistical difference in the percentage of fulfillment of RDA for iron from the water entrapped in rice and cooked rice between WDR and WSR. **No statistical difference in the percentage of fulfillment of EAR for iron from the water entrapped in rice and cooked rice between WDR and WSR. ***Recommended Dietary Allowance (RDA): Average daily level of intake sufficient to meet the nutrient requirements of nearly all (97–98%) healthy individuals.^(22)^ ****Estimated Average Requirement (EAR): Average daily level of intake estimated to meet the requirements of 50% of healthy individuals^(22)^

Figure 5(a) and (b) represent the percentage of RDA and EAR of iron fulfilled by the raw rice; water entrapped in rice and cooked rice for both WDR and WSR in the NPNL women. The consumption of raw rice (386.1 g/day) could potentially fulfil about 20.4% of RDA and 45.30% of EAR of iron in NPNL women. About 44.17% of RDA and 98.15% of EAR of iron could be met by WDR, while about 40.78% of RDA and 90.62% of EAR of iron could be achieved by WSR. Importantly, iron content in water entrapped in rice might potentially fulfil around 23.77% of RDA and 52.83% of EAR of iron by consuming cooked WDR. The iron content in water retained by rice potentially could contribute about 20.4% of the RDA and 45.30% of the EAR by the intake of cooked WSR. However, no significant difference was found between WDR and WSR in relation to the percentages of RDA and EAR fulfilled in the NPNL women.

## Discussion

The present study was carried out at Belkuchi Upazila of Sirajganj district in Bangladesh to quantify the iron in the groundwater which is entrapped in rice grains during cooking and to appraise the attribution of this novel environmental source to the dietary requirements of iron in NPNL women. The findings of the study confirmed that Belkuchi, a northern sub-district in Bangladesh, contained a high iron level in groundwater (weighted mean for water filtration was 4.99 mg/L), which is a good source of dietary iron for the people of this region. Other studies in Bangladesh highlighted the groundwater iron as an influential dietary source.^(9,10)^ In this study, the BR-28, a dominant variety of rice in Bangladesh was used and observed that the average iron content was 2.85 mg/300 g of raw rice, which was nearly similar to the estimates (2.7 mg/300 g) reported in the Food Composition Table for Bangladesh.^(17)^ The slight difference in values is possibly attributed to the geographical variation. Earlier research reported geographical variation in the mineral content of rice.^(24–26)^

It is a general understanding that WSR has a higher mineral content than WDR as the water is not removed in WSR. Interestingly, we observed significantly higher iron content in the WDR than the WSR for both filtered and non-filtered water (Figure 2). The possible reason can be explained from the observation of — a) higher volume of water used in the WDR compared to WSR (1610 ml vs 1008 ml); b) higher volume of water absorbed into the raw rice in case of the WDR (648 ml vs 578 ml, Table 4). We speculate that the higher amount of water entrapped in the rice grains in case of the WDR might be the reason for higher amount of iron in WDR than in the WSR. Cooking with iron-containing groundwater resulted in two times higher iron content in cooked rice (WDR and WSR) relative to the corresponding raw rice (Table 5). On the contrary, one study reported that the iron content decreased from 0.7 mg to 0.3 mg from raw to cooked WSR.^(17)^ This discrepancy could be attributed to the frequent washing of the rice with water and longer water soaking time before cooking, which might have decreased the iron content in the cooked rice.^(27,28)^ Moreover, different cooking procedures and using different water-rice ratios might be other possible reasons for the difference in findings.

We used 300 g of raw rice to measure the intake of water-entrapped iron from the cooked rice; and reported the extent to which the iron intake fulfilled the daily requirement of adult women. However, the mean intake of rice in rural Bangladesh is around 386.1 g^(23)^; which entails that potentially higher amounts of iron can be consumed from this novel source. Consumption of WDR and WSR fulfilled around 50% of RDA and above 90% of EAR of iron for NPNL women that was two times higher than the iron content of raw rice, assuming the daily rice consumption per person in rural Bangladesh around 386.1 g. The mineral content of groundwater research has been expanding the evidence base of the contribution of the drinking groundwater sources to the iron status in the Bangladeshi population.^(5,9,12)^ Choudhury et al has shown anaemia among children was independently associated with the iron content of drinking water.^(29)^ The present study on the water-entrapped iron in cooked rice adds an additional layer to this natural phenomenon; further strengthening the environmental contribution to nutritional status, especially for iron in the Bangladeshi population. The observed findings strengthen our assumption that the iron in the water entrapped in the cooked rice would be a fair source. However, further study is needed in two aspects: (a) Increasing the present study size to conduct it in both high and low groundwater iron settings and (b) Measuring the effect of this novel source of environmental iron on body iron reserve and Hb status. Such assessments will further strengthen the case for this novel nutrient source to consider in the research and the policy planning for iron nutriture in the Bangladeshi population for a context-specific harmonised strategy for anaemia which will be judicious in benefitting and not harming to people from adverse effects of excess iron intake. In Argentina, the groundwater mineral contribution and retention to the cooked rice were examined and the findings suggested a significant retention of minerals in cooked rice.^(30)^ Hence, the contribution of iron in cooked rice from groundwater should be essentially considered during the dietary intake assessment, which may enable more accurate assessment of the iron nutriture in population, thus guiding on the possible adjustments iron supplements interventions.

According to the National Micronutrient Survey 2011–12, anaemia and IDA are 26% and 4.8%, respectively, among Bangladeshi NPNL women aged 15 to 49 years.^(5)^ Over the decades, the government has implemented iron supplementation programme to reduce the burden of IDA in Bangladesh, targeting the high-risk populations, such as pregnant women, children and adolescents. The association between groundwater iron and haemoglobin and serum ferritin is observed in children and women.^(5,9–12,29)^ However, there is a marked heterogeneity in the iron content (low and high) in groundwater across Bangladesh. Consequently, blanket supplementation can cause an overdose of iron in those who have been exposed to high groundwater iron.^(9,16)^ Despite the beneficial effects, chronic iron overload can have several negative effects, such as diarrhoea, nausea, vomiting, and respiratory tract infection.^(13,14,31)^ On top of this, the present study demonstrates an additional layer of groundwater-entrapped iron in cooked rice. Hence, taking in consideration of iron-related side effects, a robust ‘iron mapping’ denoting differential groundwater iron for different geographical locations is required before customising the national iron supplementation programmes, so much so that the benefits of iron intervention may be maximised and harms may be limited. Further, underscoring the contribution of iron from the water entrapped in cooked rice as well as from the drinking groundwater, these sources should be integrated into the dietary assessment studies. This is likely to provide valuable insights to adjust dietary guidelines as well as iron supplementation strategies.

This study has a few limitations. A small sample size may produce a large standard error (SE) and limit the assessment of the inter-variability of the participants of the study site. Additionally, considering only high iron areas might limit the generalizability of the findings across the country, especially in the low iron areas. The study did not measure the participants’ iron status (i.e. serum ferritin concentration) and Hb to examine the association between water entrapped iron in cooked rice and iron and Hb concentration.

Cooking rice is accomplished through boiling water. When water is boiled, the dissolved Fe^2+^ iron gets oxidised to insoluble Fe^3+^ form by heating and air contact.^(32,33)^ Boiling water during rice cooking somewhat facilitates the oxidation predisposing the Fe^2+^ iron to precipitate as Fe^3+^ out of the water solution. Conversely, boiling decreases the solubility of dissolved oxygen (DO) in water by driving out the dissolved gases, thus neutralising the precipitation effect to some extent.^(34)^ Despite this boiling-induced precipitation and possible neutralisation of the phenomenon, some degree of aeration during cooking may happen. This aeration may oxidise the soluble Fe^2+^ iron to insoluble Fe^3+^ form, thus precipitating it. Interestingly, the hydrochemistry of groundwater in Bangladesh is endowed with dissolved organic mass (DOM). The oxidised Fe^3+^ iron compounded with ferrihydrite acts as colloid being stabilised by the DOM, such as fulvic acid and humic acid,^(35)^ thus the Fe^3+^ iron remains in the water solution for a considerable amount of time. Analysis from our recent study has shown that the Fe^2+^ iron-rich groundwater when kept in the open air, the decrease in the iron concentration from baseline was only 3.1% after 2 hours of holding; and the decline was statistically non-significant (Rahman S., personal communication, October 05, 2024). Since the rice-cooking time is 20–25 minutes,^(36)^ the effect of heating and potential aeration during the cooking might have decreased the iron concentration by a negligible amount if any. Thus, the estimated amount of the water-entrapped iron during rice cooking potentially emerges as a considerable source of iron. However, not being able to measure the iron concentration of boiling water during cooking was a limitation of the study. As a generic guideline, the participants were instructed to cook rice by both the WSR and WDR methods—two predominant methods of cooking in Bangladesh. We did not provide specific recipes but allowed their usual practice to cook both types. The intent was the results to be representative of the common cooking practices. This might induce potential bias to the amount of iron in the entrapped water of the cooked rice. However, individual variations in cooking might influence the amount of iron either way, thus cancelling out the effect. Nonetheless, non-provision of the specific recipes of the cooking is a limitation.

## Conclusion

Rice is a staple food for Bangladesh and the South and South-East Asian countries. Concurrently many of the countries in the region, especially in Bangladesh, there is a good level of iron in the drinking groundwater source. This study measured the iron content in a novel, unexplored natural source - the water entrapped in rice during cooking. The study observed that uses of groundwater in non-filtered forms for cooking, irrespective of the cooking methods, retained a satisfactory level of iron in rice for consumption. Irrespective of the type of cooking, the water-entrapped iron in rice fulfilled nearly a quarter of the RDA and more than half of the EAR amounts in NPNL women. Thus, this source offers substantial iron for population consumption and potentially contributing to control ID in these communities. However, small sample size of the study in a single setting is the limitations precluding optimum accuracy and generalisation of the results. Larger research across the settings would enhance the accuracy and generalisation of the phenomenon and pave the way for an optimum anaemia control policy and programme in Bangladesh.

## Data Availability

The data can be made available to readers upon a reasonable request.
